# Cardiological health in patients with schizophrenia. A prospective cohort study

**DOI:** 10.1192/j.eurpsy.2021.429

**Published:** 2021-08-13

**Authors:** R. Ernst Nielsen, M. Rodrigo-Domingo, L. Jørgensen, C. Tranekær Hostrup, S. Eggert Jensen

**Affiliations:** 1 Department Of Clinical Medicine, Aalborg University, Aalborg, Denmark; 2 Psychiatry, Aalborg University Hospital, Aalborg, Denmark

**Keywords:** Cohort, Mortality, Cardiology, schizophrénia

## Abstract

**Introduction:**

Patients with schizophrenia have a four-fold increased all-cause and a doubled cardiovascular mortality rate as compared to the general population.

**Objectives:**

The study overall investigates the point-prevalence and prospective changes in cardiovascular risk factors in patients with schizophrenia, with baseline demographics of participants presented here.

**Methods:**

A prospective study of patients diagnosed with schizophrenia divided into two subpopulations consisting of newly diagnosed (≤2 years from baseline in study (group A)) or chronic (diagnosed ≥10 years from baseline in study (group B)).

**Results:**

A total of 199 patients (57 diagnosed ≤2 years preceding baseline and 142 diagnosed ≥10 years ago) were included. Group A had been diagnosed for an average of 1.13±0.58 years and 21.19±7.62 years in group B. The majority (n=135 (67.8%)) were diagnosed with paranoid schizophrenia. At baseline PANSS total (median[Q1;Q3]) for group A was 61.0[51.0;76.0] and 60.0[48.0;76.0] for group B, with PANNS Positive being 17.0[13.0;20.0] and 15.0[12;19], PANSS Negative being 16.0[11.0;20.0] and 14.5[10.0;20.0], and PANSS General being 28.0[22.0;35.0] and30.0 [25.0;37.0], respectively. No difference in Clinical Global Impression was observed between groups ((median[Q1;Q3): 4.0[3.0;4.0] in both groups). Lastly, global assessment of function was similar between groups ((median[Q1;Q3): group A symptom: 38.5[37.0;46.0] and group B 41.0[37.0;52.0], and with function being 48.0[44.5;53.5] in group A and 45.5[41.0;53.0] in group B).

**Conclusions:**

Prospective studies investigating prevalence of and prospective changes in cardiovascular risk in patients with schizophrenia are essential to understand the increased all-cause and cardiovascular specific mortality. Demographic descriptions of participants are essential to estimate generalizability in different treatment settings.

**Disclosure:**

No significant relationships.

